# Bezafibrate for primary biliary cholangitis: A number needed to treat analysis

**DOI:** 10.1016/j.jhepr.2026.101926

**Published:** 2026-06-10

**Authors:** Ellen Werner, Maria A. van de Vrie, Maria C.B. van Hooff, Bettina E. Hansen, Adriaan J. van der Meer, Dutch PBC Study Group, Sunje Abraham, Rob P.R. Adang, Huseyin Aktas, Yasser A. Alderlieste, Maria N. Aparicio-Pages, L. (Bert) C. Baak, Martine A.M.C. Baven-Pronk, A. (Sander) van der Beek, Frank C. Bekkering, Jeroen D. van Bergeijk, Ulrich Beuers, Koen Beukema, Menno Beukema, Tiki D. Blom, Wink de Boer, Femke Boersma, Kirsten Boonstra, Frank ter Borg, Martijn J. ter Borg, Pieter C.J. ter Borg, Gijs J. de Bruin, Paul J. Bus, Djuna L. Cahen, Marcel Cazemier, Frans J.C. Cuperus, Lisette J.H. van Dam, Maaike J. Denters, Remco van Dijk, Joost P.H. Drenth, Ludger S.M. Epping, Nicole S. Erler, Hajo J. Flink, Philip W. Friederich, Nicole F.M. van Gerven, Tom J.G. Gevers, Bettina E. Hansen, Sven J. van den Hazel, Bart van Hoek, Maria C. van Hooff, Daphne M. Hotho, Harry L.A. Janssen, Hendrik J.M. de Jonge, Matthias C. Jurgens, J. (Netty) van Kemenade, Marjo J. Kerbert-Dreteler, Michael Klemt-Kropp, Ingrid C.A.W. Konings, Sander de Kort, Matthijs Kramer, Edith M.M. Kuiper, Johan P.H. Kuyvenhoven, Adriaan J. van der Meer, Suzanne van Meer, Susanne L. Onderwater, Leendert H. Oterdoom, Cyriel Y. Ponsioen, Paul G. van Putten, Janne E. van Rooij, Robert Roomer, Johannes Schmidt-Böhmer, Stephan Schmittgens, Tim C.M.A. Schreuder, Jerome Sint Nicolaas, Hanneke van Soest, Khalida Soufidi, Stephan H.C. van Stiphout, Hans H.K. Thio, Merel M. Tielemans, Sigrid Vandebosch, Rozanne C. de Veer, Bart J. Veldt, Robert C. Verdonk, J. Marleen de Vree, Elsemieke de Vries, Anne Vrieze, Jan Maarten Vrolijk, Laurens A. van der Waaij, Gemma X. Weijsters, Ellen Werner, Ulrike de Wit, Frank H.J. Wolfhagen

**Affiliations:** 1 Department of Gastroenterology and Hepatology, Erasmus University Medical Center, Rotterdam, the Netherlands; 2 IHPME, University of Toronto; Toronto, Canada; 3 Toronto Center for Liver Disease & TGHRI, University Health Network, Toronto, Canada; 4 Department of Epidemiology, Erasmus University Medical Center, Rotterdam, the Netherlands

**Keywords:** Primary Biliary Cholangitis, Second-line Therapy, Number Need to Treat

## Abstract

**Background & Aims:**

The clinical benefit of bezafibrate (BZF) in primary biliary cholangitis (PBC) has not been extensively quantified using absolute measures. We assessed the number needed to treat (NNT) with BZF to prevent liver transplantation (LT) or death (including liver-related death) in patients with PBC.

**Methods:**

The NNT was calculated using LT-free survival probabilities among ursodeoxycholic acid (UDCA)-treated patients with PBC from the Dutch PBC Cohort Study and the reported adjusted hazard ratios (aHRs) for the effect of BZF on LT or death (aHR 0.33) and liver-related death (aHR 0.27) from the Japanese cohort study (Tanaka *et al.*, J Hepatol 2021). The NNT to prevent one LT, death, or liver-related death within 5 years (NNT_5y_) or 10 years (NNT_10y_) was estimated.

**Results:**

In total, 2,271 (63.7%) patients with PBC had an alkaline phosphatase (ALP) level above the upper limit of normal (ULN) after 1 year of UDCA treatment. The median age was 57.1 years (IQR 50.1–67.0), 2,003 (88.2%) were female, and 187 (8.2%) had cirrhosis. Overall, the estimated NNT_5y_ and NNT_10y_ for BZF were 15 (95% CI 11–26) and 7 (95% CI 6–13), respectively, to prevent one LT or death, and 26 (95% CI 18–56) and 15 (95% CI 11–30), respectively, to prevent one LT or liver-related death. According to ALP category at one year of UDCA treatment (1.0–1.5 × ULN, 1.5–3.0 × ULN, and ≥3.0 × ULN), the NNT_5y_ to prevent one LT or death was 24 (95% CI 16–48), 12 (95% CI 9–23), and 7 (95% CI 5–15), respectively, while the corresponding NNT_10y_ values were 10 (95% CI 7–18), 7 (95% CI 5–13), and 4 (95% CI 3–8).

**Conclusions:**

The projected efficacy of BZF, expressed as the NNT to prevent hard clinical endpoints, was substantial overall, including among patients with relatively favourable ALP levels (1.0–1.5 × ULN) after one year of UDCA treatment. These findings highlight the potential clinical benefit of off-label BZF in UDCA-treated patients without ALP normalisation.

**Impact and implications:**

Although infrequently used in hepatology, the number needed to treat (NNT) can increase awareness among clinicians and patients about the potential impact of second-line therapy. Bezafibrate showed strong efficacy (NNT <20) for preventing liver transplantation or (liver-related) death in people with primary biliary cholangitis, even in patients with relatively favourable alkaline phosphatase levels (1.0–1.5 × the upper limit of normal) after 1 year of ursodeoxycholic acid. Findings support the potential clinical benefit of off-label bezafibrate for patients who do not achieve alkaline phosphatase normalisation on ursodeoxycholic acid alone. The NNT facilitates shared decision-making between patients and physicians on the use of second-line therapy in people with primary biliary cholangitis.

## Highlights

• Bezafibrate shows low NNTs for preventing LT or death in UDCA-treated patients with PBC.
• NNT estimates suggest meaningful benefit of BZF for hard outcomes over 5 and 10 years.
• Benefit of BZF persists even in patients with mildly elevated ALP after UDCA.
• ALP stratification shows stronger projected BZF benefit in higher biochemical risk groups.

## Graphical abstract

**Figure:**
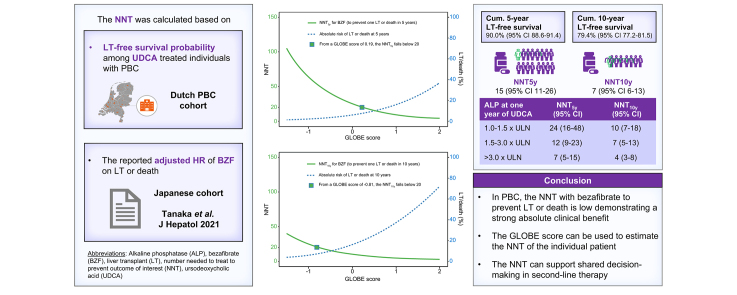

## Introduction

Primary biliary cholangitis (PBC) is a slowly progressive liver disease which can lead to cirrhosis with the risk of liver failure and hepatocellular carcinoma.^1^^,^^2^ Ursodeoxycholic acid (UDCA) is the recommended first-line therapy as it improves biochemical markers of cholestasis, slows the progression of fibrosis, and improves liver transplant (LT)-free survival.^3^^,^^4^ Still, 30-40% of people with PBC have an incomplete biochemical response after 1 year of UDCA, which is associated with impaired long-term clinical outcomes.^5^ For these individuals, add-on second-line therapy should be considered.^3^

Currently, multiple on- and off-label agonists of the peroxisome proliferator-activated receptor (PPAR) are available.^6^ These drugs have been shown to further decrease cholestatic markers, which have recently been associated with prognosis in the setting of second-line therapy as well.^7^^,^^8^ Indeed, due to long-term off-label use, bezafibrate (BZF) could be linked to a reduction in the risk of LT or death.^9^

In this report, we model the proposed clinical benefit of BZF for PBC into an easy-to-understand absolute clinical efficacy measure; the number-needed-to-treat to prevent one LT or (liver-related) death. Although infrequently reported in hepatology, such an effect measure can aid the awareness among clinicians and patients on the potential impact of second-line therapy.

## Patients and methods

To calculate the NNT to prevent one LT or death, the LT-free survival probability with UDCA monotherapy was calculated among UDCA-treated individuals with PBC included in the Dutch PBC Cohort Study. This retrospective national cohort has been described in detail previously.^10^ The Dutch PBC Cohort Study was used as it provides recent representative estimates of outcome considering that the cohort includes all identifiable patients with PBC within the entire healthcare setting of a single country (The Netherlands). If biochemical data were available between 6 and 18 months after initiation of UDCA, the biochemical value at exactly 1 year after start of UDCA was imputed using joint model multiple imputation, which considered sex, age at UDCA initiation, and liver decompensation over time. As a sensitivity analysis, complete-case analyses were performed, including patients with available biochemical measurements closest to 1 year after UDCA initiation, provided these were obtained within a 6–18-month window.

Kaplan-Meier analyses were used to assess the cumulative LT-free survival across multiple follow-up periods, both in the overall population with an abnormal alkaline phosphatase (ALP) after 1 year of UDCA and in different ALP subgroups (in line with cut-offs in the biochemical UDCA response criteria). Further stratified survival estimates were calculated for clinically relevant subgroups. Follow-up was censored at the last follow-up visit or at the initiation of second-line therapy.

The primary analyses focused on the NNT to prevent one LT or death with BZF therapy. While the Dutch PBC cohort study provides representative real-world estimates of prognosis in UDCA-treated patients with PBC, it currently lacks sufficient unbiased data to reliably estimate the association between BZF and survival due to a relatively short follow-up in this selected group, who were already on UDCA for a median duration of 7.3 years. In Japan, however, there is long-term off-label experience with this PPAR agonist resulting in a long mean BZF exposure of 5.3 years. Moreover, the time between start of UDCA and BZF (1.4 years) in the Japanese PBC cohort was relatively short. Therefore, Tanaka *et al.* provides the most reliable estimate on the association between BZF and long-term clinical outcome in PBC to date.^9^ To model the NNT with BZF in our Western population, the adjusted hazard ratio (HR) estimates for BZF from the Japanese study were used; this was 0.33 (95% CI 0.19-0.55) for LT or death and 0.27 (95% CI 0.13-0.57) for LT or liver-related death. The confidence interval was incorporated into the uncertainty of the NNT estimates.

The NNT was calculated using the following formula:

$${\text{NNT}}_{\left(t\right)}=\left(1/\left(\text{LT}-\text{free}\;\text{survival}\;{{\text{UDCA}}_{\left(t\right)}}^{\text{adjusted}\;\text{HR}\;\text{BZF}}-\mathrm{LT}-\text{free}\;\text{survival}\;{\text{UDCA}}_{\left(t\right)}\right)\right)\text{.}$$

The NNT to prevent one event can be calculated for every follow-up period of interest, corresponding to the appropriate cumulative event rate at that specific follow-up time. As the event rate increased over time, the NNT reduces in case one event is to be prevented over a longer period. Here, we focus on the NNT to prevent one event at 5 years (NNT_5y_) and at 10 years (NNT_10y_).

In addition, considering that LT-free survival can be accurately estimated with the validated objective GLOBE score, the NNT was plotted over the range of the GLOBE score to enable estimation of the NNT to prevent one LT or death for the individual patient.

## Results

In total, 2,271 (63.7%) people with PBC had an ALP above the ULN after 1 year of UDCA therapy. At that time, median age was 57.1 years (IQR 50.1–67.0), 2,003 (88.2%) were female, and 187 (8.2%) had cirrhosis. During a median follow-up of 7.5 years (IQR 3.5–13.2), 76 patients underwent LT and 411 patients died (without LT). Overall, the cumulative 5-year and 10-year LT-free survival was 90.0% (95% 88.6–91.4) and 79.4% (95% CI 77.2–81.5), respectively. This corresponded to an estimated overall NNT_5y_ of 15 (95% CI 11–26) and an overall NNT_10y_ of 7 (95% CI 6–13) to prevent one LT or death with BZF therapy in the population with an elevated ALP after at least 1 year of UDCA therapy. The complete case analysis yielded the same overall NNT estimates. Table 1 shows the estimated NNT_5y_ and NNT_10y_ in various subgroups. Among patients with an ALP >1.5 × ULN, who are generally considered for second-line therapy, the NNT_5y_ and NNT_10y_ were 11 (95% CI 8–19) and 6 (95% CI 4–10), respectively. Among those with an ALP between 1.0 × ULN and 1.5 × ULN, representing a subgroup in whom second-line therapy may now be initiated as normal ALP has been associated with the best clinical outcome, the NNT_5y_ and NNT_10y_ were 24 (95% CI 16–48) and 10 (95% CI 7–18), respectively. For LT or liver-related death, the overall NNT_5y_ and NNT_10y_ with BZF were 26 (95% CI 18–56) and 15 (95% CI 11–30), respectively. Table 2 shows that the NNTs of BZF are somewhat higher for this secondary endpoint in all subgroups of patients, in line with the lower event rates at both 5 years and 10 years of follow-up. All NNT results were consistent in the complete case analyses (data not shown).

**Table 1:** The cumulative survival in people using ursodeoxycholic acid monotherapy and corresponding number needed to treat with add-on bezafibrate in the population with primary biliary cholangitis.

	Cumulative 5-year LT-free survival (95% CI)	Estimated NNT_5y_ (95% CI)	Cumulative 10-year LT-free survival (95% CI)	Estimated NNT_10y_ (95% CI)
ALP >1.0 × ULN	90.0% (88.6–91.4)	15 (11–26)	79.4% (77.2–81.5)	7 (6 -13)
ALP 1.0-1.5 × ULN	93.8% (92.2–95.3)	24 (16–48)	84.2% (81.5–86.9)	10 (7–18)
ALP >1.5-3.0 × ULN	87.7% (85.2–90.3)	12 (9–23)	78.1% (74.6–81.8)	7 (5–13)
ALP >1.5 × ULN	85.5% (83.2–88.0)	11 (8–19)	73.9% (70.6–77.3)	6 (4–10)
ALP >3.0 × ULN	78.5% (72.9–84.5)	7 (5 -15)	60.8% (53.7–68.8)	4 (3–8)
Bilirubin ≤1 × ULN	94.5% (93.2–95.7)	27 (18–52)	84.2% (81.9–86.4)	10 (7–17)
Bilirubin >1 × ULN	57.8% (51.3–65.1)	4 (3–7)	43.8% (37.0–51.7)	3 (2–6)
Age ≤55	94.6% (93.0–96.3)	28 (18–60)	90.0% (87.6–92.5)	15 (10–30)
Age >55	86.7% (84.6–88.8)	11 (8–20)	71.8% (68.7–75.0)	6 (4–10)
No cirrhosis	93.0% (91.7–94.2)	21 (15–39)	83.4% (81.4–85.5)	9 (7–16)
Cirrhosis	55.2% (47.6–63.9)	4 (3–7)	31.8% (24.1–41.9)	3 (2–5)

ALP, alkaline phosphatase; LT, liver transplant; NNT, number needed to treat; ULN, upper limit of normal.

The cumulative survival was estimated using the Kaplan–Meier method.

**Table 2:** The cumulative survival free of liver transplantation or liver related death in people using ursodeoxycholic acid monotherapy and corresponding number needed to treat with add-on bezafibrate in the population with primary biliary cholangitis.

	Cumulative 5-year LR-free survival (95% CI)	Estimated NNT_5y_ (95% CI)	Cumulative 10-year LR-free survival (95% CI)	Estimated NNT_10y_ (95% CI)
ALP >1.0 × ULN	94.8% (93.7–95.8)	26 (18–56)	90.5% (88.9–92.1)	15 (11–30)
ALP 1.0-1.5 × ULN	97.4% (96.3–98.4)	53 (31 -149)	95.3% (93.7–96.9)	29 (18–75)
ALP >1.5-3.0 × ULN	94.6% (92.8–96.4)	26 (16–64)	89.4% (86.7–92.2)	13 (9–30)
ALP >1.5 × ULN	91.7% (89.8–93.7)	17 (11–37)	85.1% (82.4–87.9)	9 (7–20)
ALP >3.0 × ULN	82.5% (77.2–88.2)	8 (5–20)	71.9% (65.1–79.4)	5 (3–12)
Bilirubin ≤1 × ULN	98.7% (98.1–99.3)	105 (60–337)	95.5% (94.2–96.9)	31 (20–74)
Bilirubin >1 × ULN	63.5% (56.8–70.9)	4 (3–9)	52.4% (45.1–60.7)	3 (2–7)
Age ≤55	95.1% (93.6–96.7)	28 (18–70)	91.7% (89.5–93.9)	17 (11–38)
Age >55	94.5% (93.0–95.9)	25 (17–57)	89.6% (87.4–91.9)	13 (9–29)
No cirrhosis	97.3% (96.4–98.1)	51 (33–122)	94.1% (92.7–95.5)	24 (16–51)
Cirrhosis	62.9% (54.8–72.0)	4 (3–9)	43.1% (33.6–55.3)	3 (2–6)

ALP, alkaline phosphatase; LR, liver-related death; NNT, number needed to treat; ULN, upper limit of normal.

The cumulative survival was estimated using the Kaplan–Meier method.

**Fig. 1:**
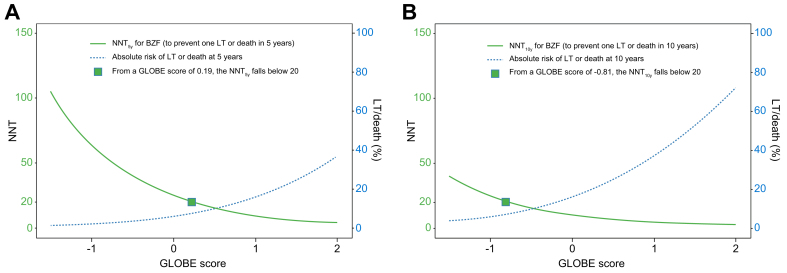
The number needed to treat for add-on bezafibrate and risk of liver transplant or death according to the GLOBE score at 1 year after start of ursodeoxycholic acid. Figure shows the NNT with bezafibrate as second-line therapy *vs*. the cumulative risk of LT or death, based on the GLOBE score in the population with PBC. From a GLOBE score of 0.19, the NNT_5y_ falls below 20 (A), which was -0.81 for the NNT_10y_ (B). BZF, bezafibrate; LT, liver transplant; NNT, number needed to treat. The cumulative survival was estimated using the Kaplan–Meier method.

As Table 1 reflects that the NNT depends on the absolute risk of LT of death, we next assessed the NNT according to the validated GLOBE score which objectively estimates the individual patient’s risk of LT or death with UDCA monotherapy. In addition to the risk of LT or death, Fig. 1 plots the NNT according to the GLOBE score. As visualized, the lower the GLOBE score (and thus the better the LT-free survival), the higher the NNT. The NNT_5y_ and NNT_10y_ dropped below 20 from a GLOBE score above 0.19 and –0.81, respectively, corresponding to an estimated cumulative LT-free survival of 92.3% at either 5 or 10 years. The NNT_5y_ was below 20 for 768/1,741 (44.1%) people in the analyses and the NNT_10y_ was below 20 for 1,494/1,741 (85.8%) people with PBC and an abnormal ALP at 1 year of UDCA.

## Discussion

In the population with PBC in need of second-line therapy, our results show that the NNT to prevent one LT or (liver-related) death with BZF is generally low. The higher the ALP level after 1 year of UDCA, the stronger the absolute clinical efficacy of BZF. After 5 years of add-on BZF therapy, the NNT to prevent one LT or death was 15, and this number decreased to 7 when estimating prevention over 10 years. To prevent one LT or liver-related death, 26 individuals need to be treated for 5 years, and 15 individuals for 10 years. Even among patients with relatively favourable biochemical response to 1 year of UDCA, those with an elevated ALP but below 1.5 × ULN, the NNT_10y_ of 10 (95% CI 7-18) to prevent one LT or death can be considered low. Based on the GLOBE score, the NNT_10y_ to prevent one LT or death was below 20 for more than 85% of patients with an ALP above the ULN after 1 year of UDCA monotherapy. Thus, these results suggest strong clinical efficacy of BZF and may encourage its use as off-label second-line therapy in PBC.

The NNT was assessed in both the overall cohort and relevant subgroups based on various cut-offs of biochemical markers of disease severity. The lowest NNTs were observed for people with cirrhosis, although caution is advised when considering second-line therapy in this subgroup. Substantially lower NNTs were also seen in those with higher levels of ALP and total bilirubin. This can be explained by the worse LT-free survival in these individuals and underscores the need to consider the clinical setting when evaluating the benefit of treatment. This is indeed integrated in the NNT, while lacking with more common relative measures of risk reduction such as the hazard ratio. Broadening our understanding of the clinical benefit of anticholestatic therapy for PBC is important. It has been shown that physicians tend to overestimate the benefit of treatment when relying on measures of relative risk reduction.^11^ According to the GLOBE score, our results show that the majority (85%) of patients with persistent ALP elevation after 1 year of UDCA have a strong absolute clinical benefit from BZF add-on therapy (NNT_10y_ to prevent one LT or death <20). For others with a more favourable prognosis during UDCA monotherapy, the NNT will probably reduce if an event is to be prevented over longer periods of therapy. While carefully weighing the potential benefits of off-label BZF therapy against the risk of adverse effects, particularly in patients with more advanced liver disease, these relatively low NNTs may facilitate shared decision-making when considering second-line therapy. To place our estimates into perspective, therapeutic drugs such as statins are prescribed even at a NNT greater than 200 within a 5-year time window.^12^

In the study of Tanaka *et al.*, an overall NNT of 29 (95% CI 22-46) at 5 years and 14 (95% CI 10-22) at 10 years was reported to prevent one LT or death with BZF.^9^ This is somewhat higher as presented here, which may be explained by differences in methodology. To assess the overall event-free survival within patients unexposed to BZF, we restricted our assessment to those patients with persistent cholestasis with UDCA alone. This is not specified for the Japanese study. However, including patients with normal ALP following UDCA initiation (about one-third of people) positively influences the overall survival estimate, which increases the NNT. Another factor to consider is that the baseline mean ALP of 3.2 × ULN (SD 2.3) in our cohort was substantially higher than that in the Japanese cohort (mean ALP of 2.31 × ULN, SD 1.90). However, a relevant message based on our results is that there is not a single estimate of the NNT with BZF. Rather, the NNT varies per individual patient based on his or her risk of the outcome of interest. The visualisation of the differences in the NNT between various patient risk groups may therefore be more relevant than the actual estimates per subgroup itself. This limits the relevance of the comparison of overall estimates in different populations and is also why we included an assessment of the NNT in relation to the GLOBE score. This approach provides a NNT estimate per individual patient based on his or her estimated risk.

Nevertheless, a limitation of this study is that the estimated relative risk reduction associated with BZF was derived from a single Japanese PBC cohort with long-term follow-up, which may limit the generalisability of these findings to other populations. To improve extrapolation, we used the adjusted HR of BZF and we have taken the confidence intervals of the relative risk reduction estimate into account. It may be debated whether the overall adjusted HR reported by Tanaka *et al.* is appropriate for calculating NNTs in different subgroups. However, in line with the results for UDCA, the Japanese cohort showed consistent relative risk reductions with BZF across relevant patient subgroups.^9^^,^^13^ The variation in NNT appears to be mainly driven by differences in survival probability rather than by small differences in treatment effect. Together, we believe that the overall adjusted HR is sufficiently robust for estimating the NNT in our subgroups of interest. Lastly, as Tanaka *et al.* may not have restricted their comparator population to those with persistent ALP elevation with UDCA alone (thereby favouring the prognosis in their control arm), their estimated association between BZF and outcome may be underestimated. This would, however, imply that our NNT estimates are conservative.

How our results translate to the new, more selective PPAR agonists, which have emerged and can already be prescribed in various countries, is currently unknown.^14^^,^^15^ Long-term follow-up data of people who initiated these new drugs are not yet available. Interestingly, obeticholic acid resulted in similar HRs as BZF with respect to LT or all-cause mortality, so it may be anticipated that the absolute clinical benefit of seladelpar and elafibranor also falls within the confidence intervals of the estimates we presented here.^16^^,^^17^

To conclude, our results reinforce the strong clinical benefit of off-label BZF in patients with PBC and persisting cholestasis despite UDCA monotherapy. The GLOBE score after 1 year of UDCA, and the corresponding survival probability, allows for the estimation of an individual’s NNT. This may help guide personalized treatment decisions.

## Abbreviations

ALP, alkaline phosphatase; BZF, bezafibrate; HR, hazard ratio; LT, liver transplant; NNT, number needed to treat; PBC, primary biliary cholangitis; PPAR, peroxisome proliferator-activated receptor; UDCA, ursodeoxycholic acid; ULN, upper limit of normal.

## Author contributions

Study concept and design: EW, MV, MH, BH, AM. Data acquisition: all authors. Data Analysis: EW, MV, MH, AM. Data Interpretation: EW, MV, MH, AM, BH. Drafting manuscript: EW, MV, MH, AM. Critical revision for important intellectual content and final approval: EW, MV, MH, BH, AM.

## Data availability

As this is a multicenter study in which data among 71 centers is shared, the dataset which was generated is not openly available for researchers outside of the study team. Please contact the corresponding author to inquire about the possibilities of collaboration.

## Declaration of generative AI and AI-assisted technologies in the writing process

During the preparation of this work, the author(s) used ChatGPT to improve the clarity and quality of the written content. After using this tool, the author(s) carefully reviewed and edited the material as necessary and take full responsibility for the content of the publication.

## Financial support

No financial support was provided for this study.

## Conflicts of interest

Bettina E. Hansen received grants from: Mirum, Intercept Pharmaceuticals, Gilead Sciences, and Ipsen; is consultant for Mirum, Intercept Pharmaceuticals, Gilead Sciences, Ipsen, Advanz Pharma, Falk and Arbutus. Adriaan J. van der Meer received grants from: CymaBay Therapeutics, Intercept Pharmaceuticals, Gilead Sciences, MSD, Mirum and Zambon Nederland B.V; is consultant for Intercept Pharmaceuticals, Advanz Pharma, AOP Health, CymaBay Therapeutics, and Ipsen, and receives speakers fee from Zambon Nederland B.V. and AOP Health. All other authors report no potential conflict of interest for this manuscript.

Please refer to the accompanying ICMJE disclosure forms for further details.

## Appendix

### Collaborators

All collaborating authors of the **Dutch PBC Study Group**: Sunje Abraham; Rob P.R. Adang; Huseyin Aktas; Yasser A. Alderlieste; Maria N. Aparicio-Pages; L. (Bert) C. Baak; Martine A.M.C. Baven-Pronk; A. (Sander) van der Beek; Frank C. Bekkering; Jeroen D. van Bergeijk; Ulrich Beuers; Koen Beukema; Menno Beukema; Tiki D. Blom; Wink de Boer; Femke Boersma; Kirsten Boonstra; Frank ter Borg; Martijn J. ter Borg; Pieter C.J. ter Borg; Gijs J. de Bruin; Paul J. Bus; Djuna L. Cahen; Marcel Cazemier; Frans J.C. Cuperus; Lisette J.H. van Dam; Maaike J. Denters; Remco van Dijk;Joost P.H. Drenth; Ludger S.M. Epping; Nicole S. Erler; Hajo J. Flink; Philip W. Friederich; Nicole F.M. van Gerven; Tom J.G. Gevers; Bettina E. Hansen; Sven J. van den Hazel; Bart van Hoek; Maria C. van Hooff; Daphne M. Hotho; Harry L.A. Janssen; Hendrik J.M. de Jonge; Matthias C. Jurgens; J.(Netty) van Kemenade; Marjo J. Kerbert-Dreteler; Michael Klemt-Kropp; Ingrid C.A.W Konings; Sander de Kort; Matthijs Kramer; Edith M.M. Kuiper; Johan P.H. Kuyvenhoven; Adriaan J. van der Meer; Suzanne van Meer; Susanne L. Onderwater; Leendert H. Oterdoom; Cyriel Y. Ponsioen; Paul G. van Putten; Janne E. van Rooij; Robert Roomer; Johannes Schmidt-Böhmer; Stephan Schmittgens; Tim C.M.A. Schreuder; Jerome Sint Nicolaas; Hanneke van Soest; Khalida Soufidi; Stephan H.C. van Stiphout; Hans H.K. Thio; Merel M. Tielemans; Sigrid Vandebosch; Rozanne C. de Veer; Bart J. Veldt; Robert C. Verdonk; J. Marleen de Vree; Elsemieke de Vries; Anne Vrieze; Jan Maarten Vrolijk; Laurens A. van der Waaij; Gemma X. Weijsters; Ellen Werner; Ulrike de Wit; Frank H.J. Wolfhagen.
