# Supplementary material for: Bezafibrate for primary biliary cholangitis: A number needed to treat analysis
Source: JHEP Rep. 2026 Jun 10;8(8):101926. doi: 10.1016/j.jhepr.2026.101926 (PMC13382791; doi:10.1016/j.jhepr.2026.101926)
Supplement: Multimedia component 1 [file mmc1.docx]

**JHEP Reports**

**CTAT methods**

Tables for a “Complete, Transparent, Accurate and Timely account” (CTAT) are now mandatory for all revised submissions. The aim is to enhance the reproducibility of methods.

- Only include the parts relevant to your study
- Refer to the CTAT in the main text as ‘Supplementary CTAT Table’
- Do not add subheadings
- Add as many rows as needed to include all information
- Only include one item per row

**If the CTAT form is not relevant to your study, please outline the reasons why:**

| For our manuscript “Bezafibrate for Primary Biliary Cholangitis: a Number Needed to Treat Analysis”, we used an existing hazard ratio published by Tanaka et al. We only calculated the survival probability within the Dutch PBC Cohort using UDCA monotherapy with ALP levels above 1.0 after one year of UDCA. It concerns an observational study. We kindly refer to 1.7 Software and 1.9 (details corresponding author). . |
| --- |

- 1. **Antibodies**

| **Name** | **Citation** | **Supplier** | **Cat no.** | **Clone no.** |
| --- | --- | --- | --- | --- |
|  |  |  |  |  |

- 1. **Cell lines**

| **Name** | **Citation** | **Supplier** | **Cat no.** | **Passage no.** | **Authentication test method** |
| --- | --- | --- | --- | --- | --- |
|  |  |  |  |  |  |

- 1. **Organisms**

| **Name** | **Citation** | **Supplier** | **Strain** | **Sex** | **Age** | **Overall n number** |
| --- | --- | --- | --- | --- | --- | --- |
|  |  |  |  |  |  |  |

- 1. **Sequence based reagents**

| **Name** | **Sequence** | **Supplier** |
| --- | --- | --- |
|  |  |  |

- 1. **Biological samples**

| **Description** | **Source** | **Identifier** |
| --- | --- | --- |
|  |  |  |

- 1. **Deposited data**

| **Name of repository** | **Identifier** | **Link** |
| --- | --- | --- |
|  |  |  |

- 1. **Software**

| **Software name** | **Manufacturer** | **Version** |
| --- | --- | --- |
| RStudio | R Foundation for Statistical Computing, Vienna, Austria | 4.5.2 |

- 1. **Other (*e.g*. drugs, proteins, vectors etc.)**

|  |  |  |
| --- | --- | --- |
|  |  |  |

- 1. **Please provide the details of the corresponding methods author for the manuscript:**

| Adriaan J. van der Meer, MD, PhD  Erasmus MC, University Medical Center  Department of Gastroenterology and Hepatology  Doctor Molewaterplein 40NA building, Floor 6  3015 GD Rotterdam  The Netherlands  Telephone: +31107044215  a.vandermeer@erasmusmc.nl |
| --- |

**2.0 Please confirm for randomised controlled trials all versions of the clinical protocol are included in the submission. These will be published online as supplementary information.**

|  |
| --- |
